# Nanograin network memory with reconfigurable percolation paths for synaptic interactions

**DOI:** 10.1038/s41377-023-01168-5

**Published:** 2023-05-15

**Authors:** Hoo-Cheol Lee, Jungkil Kim, Ha-Reem Kim, Kyoung-Ho Kim, Kyung-Jun Park, Jae-Pil So, Jung Min Lee, Min-Soo Hwang, Hong-Gyu Park

**Affiliations:** 1 Department of Physics, Korea University, Seoul, 02841 Republic of Korea; 2 Department of Physics, Jeju National University, Jeju, 63243 Republic of Korea; 3 Department of Physics, Chungbuk National University, Cheongju, 28644 Republic of Korea

**Keywords:** Nanowires, Photonic devices

## Abstract

The development of memory devices with functions that simultaneously process and store data is required for efficient computation. To achieve this, artificial synaptic devices have been proposed because they can construct hybrid networks with biological neurons and perform neuromorphic computation. However, irreversible aging of these electrical devices causes unavoidable performance degradation. Although several photonic approaches to controlling currents have been suggested, suppression of current levels and switching of analog conductance in a simple photonic manner remain challenging. Here, we demonstrated a nanograin network memory using reconfigurable percolation paths in a single Si nanowire with solid core/porous shell and pure solid core segments. The electrical and photonic control of current percolation paths enabled the analog and reversible adjustment of the persistent current level, exhibiting memory behavior and current suppression in this single nanowire device. In addition, the synaptic behaviors of memory and erasure were demonstrated through potentiation and habituation processes. Photonic habituation was achieved using laser illumination on the porous nanowire shell, with a linear decrease in the postsynaptic current. Furthermore, synaptic elimination was emulated using two adjacent devices interconnected on a single nanowire. Therefore, electrical and photonic reconfiguration of the conductive paths in Si nanograin networks will pave the way for next-generation nanodevice technologies.

## Abstract (web summary)

Nanograin network memory is developed using reconfigurable percolation paths in a single Si NW with solid core and porous shell, demonstrating analog adjustment of persistent current level.

## Graphical abstract

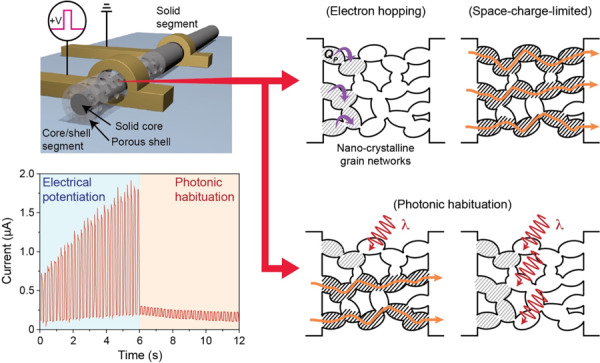

## Introduction

Simultaneous processing and storage of data in a single memory device are required for efficient computation, in addition to the traditional read and write functions in the von Neumann-structured device^1,2^. To this end, artificial synaptic devices to control signal weights have been developed by mimicking synaptic behaviors in biological systems^3–8^. While arrays of the devices are capable of neuromorphic computation, single devices alone can form hybrid networks with biological neurons that enable interaction and communication between the brain and computer^3–11^. Approaches such as filament formation and ion-transport recombination have been widely used to control the current level and perform the computation; an electric field causes filament formation or ion vacancy movement in metal oxides of memristors, thereby allowing non-volatile resistance switching and analog in-memory computation^12–18^. However, it is widely known that performance degradation of these devices is unavoidable because of the irreversible aging caused by the evolution of the internal structures^19^.

On the other hand, photonic devices have been proposed for controlling current levels without device degradation^20–24^. For example, photon-triggered transistors and atomically thin phototransistors were successfully demonstrated showing high device performance^20,21^. Photonic synapses for neuromorphic applications were also demonstrated, including the utilization of low-dimensional materials^25,26^. However, photocurrent generation in these semiconductor devices was typically used for current enhancement^20–26^. Suppression of current levels and switching of analog conductance remain challenging in a photonic manner. Although light-induced current reduction has been reported in graphene/MoS_2_ photoresponsive devices and mechano-photonic devices^27,28^, the fact that these devices require specific conditions for their operation, such as low temperatures of 130 K or mechanical displacements in millimeters, places critical constraints on their practical implementation and integration. Therefore, using the advantages of electronic and photonic devices, it is necessary to demonstrate the analog and reversible control of a persistent current path in a nanodevice without structural deformation. Such a memory device will be useful not only for simultaneous data processing and storage, but also for advanced applications such as synaptic interactions.

Here, we demonstrated reconfigurable percolation paths in nanograin networks for synaptic interactions, using a single Si nanowire (NW) with a solid core and porous shell segment. The electrical and photonic control of the conductive paths in the Si nanograin networks of the NW shell efficiently adjusted the persistent current level in an analog and reversible manner. In addition to memory behavior, photonic habituation in the NW device was demonstrated by abruptly disconnecting the current percolation path under laser illumination. Furthermore, using potentiation and habituation processes, the characteristics of a nanoscale synaptic device were demonstrated in this single NW memory. In particular, synaptic elimination was achieved in two adjacent devices interconnected on a single NW, by using photonic habituation as a kill switch for the device under illumination. We believe that electrical and photonic reconfiguration of the conductive paths in Si nanograin networks, as well as emulating synapses in NW memory, will be essential for next-generation nanodevice technologies.

## Results

**Fig. 1:**
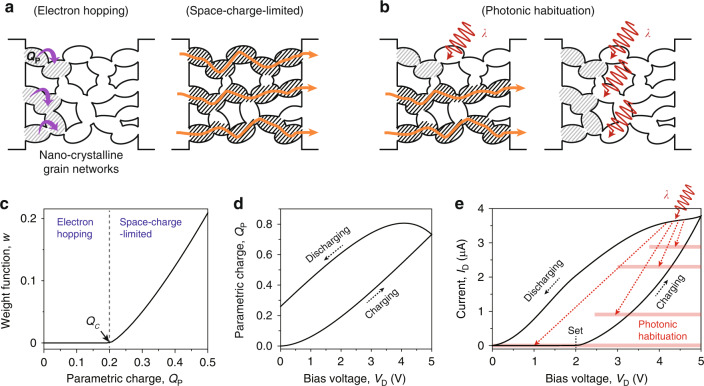
Reconfigurable current percolation paths in nanograin networks. **a**, **b** Schematic diagrams describing the current flow in the nano-crystalline grain networks. **a** Current paths are formed depending on charging in the networks. The electron hopping is dominant in the low charging condition due to the Coulomb barrier in nanograins (left). With increasing electrical charging, the current percolation paths are created and the space-charge-limited current flows (right). Gray and black slashes indicate low and high charging in the networks, respectively. **b** The current percolation paths are blocked under illumination (left). With increasing incident light intensity, more current percolation paths are disconnected (right). **c** Calculated weight function, *w*, as a function of parametric charge, *Q*_*P*_. *Q*_*C*_ is the critical parametric charge. Calculated *Q*_*P*_ (**d**) and total current *I*_*D*_ (**e**) as a function of bias voltage, *V*_*D*_. *Q*_*C*_ was set to 0.2, which corresponds to the set point at *V*_*D*_ = 2 V. Charging and discharging (black arrows) occur in the forward and backward sweeps in *V*_*D*_, respectively. Photonic habituation occurs by light illumination on the nanograin networks (red arrows), based on the operational mechanism in (**b**)

We utilize nanograin networks to control persistent current paths in a reversible manner without structural deformation (Fig. 1a, b). Because numerous nanograins are interconnected, such networks have a high resistance; however, electrical charging can form current percolation paths with a lower resistance. Indeed, electric charges are stored in the networks because of the self-capacitive nature of the nanograins^29,30^. As the electrical charges increase, the current flow process changes from electron hopping to space-charge-limited one (Fig. 1a). First, the electron hopping is dominant in the absence of charging, due to the Coulomb barrier in nanograins (left, Fig. 1a). The current percolation paths start to be created by charging in the nanograin networks, allowing for the space-charge-limited current (right, Fig. 1a). We note that the current by electron hopping (*I*_*H*_) is much lower than the space-charge-limited current (*I*_*SCL*_). This is due to the fact that the electrical connection in the percolation path is simply accomplished by the charged nanograin networks, whereas hopping process requires the activation energy to overcome Coulomb barrier^31^. Thus, by adjusting the *I*_*H*_ and *I*_*SCL*_, the analog control of the persistent current level is feasible.

The reverse process can be demonstrated by reducing electrical charging. Although a reverse bias voltage can be applied for this purpose, percolation paths in the networks are simply re-created in the backward direction, preventing an effective decrease in current. Interestingly, the nanograin networks facilitate photonic habituation that progressively suppresses the current by the annihilation of charges and the disconnection of the current percolation paths under illumination (Fig. 1b). The charges stored in the nanograin networks are released in the light condition. This process is opposed to that the current is enhanced by the photocarrier generation in semiconductors^21^. Depending on the light intensity, the current can be gradually reduced by disconnecting part or all of the percolation paths (left and right, Fig. 1b).

We theoretically investigate the contribution of *I*_*H*_ and *I*_*SCL*_ to the total current using the percolation theory of conductivity^32^. The stored charge in the nanograin networks is described by the parametric charge, *Q*_*P*_ (see “Methods” section). As a function of *Q*_*P*_, we calculated the weight function, *w*, which indicates the number of current percolation paths (Fig. 1c). Based on the percolation theory of conductivity, *w* was zero when *Q*_*P*_ < *Q*_*C*_ because there were no current percolation paths, whereas *w* increased when *Q*_*P*_ > *Q*_*C*_, where *Q*_*C*_ is the critical parametric charge. Notably, the total current, *I*_*D*_ = (1 − *w*) *I*_*H*_ + *w I*_*SCL*_, was determined by the history of the applied bias voltage, *V*_*D*_. We then calculated *Q*_*P*_ and *I*_*D*_ with the *V*_*D*_ sweep in our model (Fig. 1d, e). As *V*_*D*_ increases from 0 to 5 V (forward sweep), *Q*_*P*_ and *I*_*D*_ increase by charging. *I*_*D*_ is the same as *I*_*H*_ until the set point (*Q*_*P*_ = *Q*_*C*_), but then starts to increase rapidly with the contribution of *I*_*SCL*_ (Fig. 1e). On the other hand, *I*_*D*_ (and *Q*_*P*_) decreases for the backward *V*_*D*_ sweep; at the same *V*_*D*_, *I*_*D*_ exhibits a larger value than the one with the forward *V*_*D*_ sweep. This hysteresis loop is formed due to the delayed response of *Q*_*P*_ in the *V*_*D*_ sweep. Since the characteristic time for charging in the nanograin networks is an order of seconds by the high resistance of hopping transport^31^, the charging (or discharging) does not immediately follow the *V*_*D*_ sweep. Moreover, the quick analog suppression of *I*_*D*_ under illumination can erase the history of the applied *V*_*D*_ (red arrows, Fig. 1e), providing a function of reversible kill switch of charging.

**Fig. 2:**
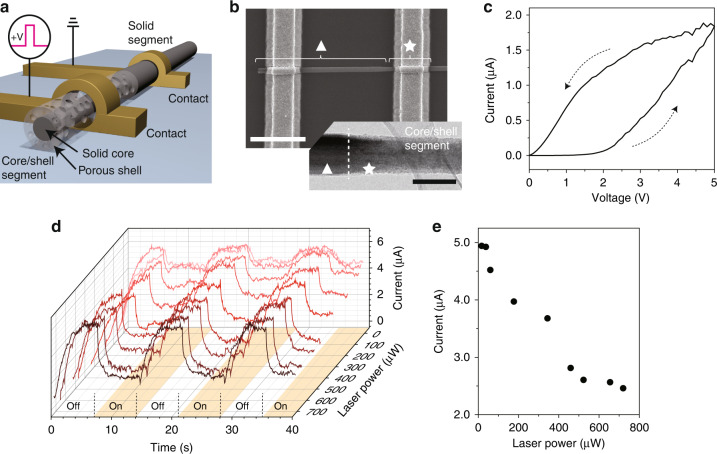
Memory behavior and photonic habituation in a NW device. **a** Schematic of the Si NW consisting of the solid core/porous shell and pure solid core segments. Two electrodes are placed on each segment. **b** SEM image of a fabricated NW device. Symbols of ▲ and ★ indicate the pure solid and core/shell segments, respectively. Scale bar, 2 μm. Lower inset, TEM image of the interface between the pure solid and core/shell segments (white dashed line). Scale bar, 200 nm. **c** Measured *I*–*V* curve for the NW device with a dual sweep mode. The black dotted arrows indicate the sweep direction. **d** Measured currents as a function of time, under the 658-nm laser illumination with powers of 17, 38, 60, 177, 334, 459, 523, 655, and 720 μW. The pump laser cycled between the on (orange region) and the off states for every 7 s. **e** Measured currents at 40 s in (**d**) as a function of the laser power

These unique properties of nanograin networks can be realized using porous Si structures on the tens of nanometer scale^20^. The memory device is specifically implemented by rationally designing a single Si NW with a solid core and porous shell structure (Fig. 2a). This Si NW has two distinct segments along the longitudinal NW axis. One segment is composed of the single-crystal solid Si core and the porous Si shell, whereas the other is only the solid Si. Two electrodes are placed on each segment to apply the bias voltage to the porous shell. Then, the proposed memory property can be seen between the solid core channel and the electrode.

To fabricate the NW devices with two structurally distinct segments, we used metal-assisted chemical etching (see “Methods” section)^20^. As shown in a scanning electron microscope (SEM) image of a NW structure (Fig. 2b), the left and right electrodes were fabricated on the long solid segment (▲) and the short core/shell segment (★), respectively. In addition, we performed the transmission electron microscopy (TEM) analysis to investigate the interface between the pure solid and core/shell segments (inset, Fig. 2b). The ~15-nm-thick porous shell was observed in the core/shell segment, showing a brighter contrast than the solid core. This feature was more clearly seen in the high-resolution TEM image, in which the core/shell segment exhibited numerous nanometer-sized Si crystallite networks and nanoscale voids of the porous shell and single-crystal lattice of the solid core (Fig. S1).

To investigate the memory property, we measured the *I*–*V* curve of the fabricated NW device. The current changed by dual sweeping of applied voltage (black arrows) (Fig. 2c). A clear hysteresis loop was observed at bias voltages between 0 and 5 V, as expected in the theoretical analysis of Fig. 1e, whereas the pure Si NW device exhibited a linear *I*–*V* curve (Fig. S2). The current showed a similar behavior in the negative voltage sweep, but the lower current level was formed because of the Schottky barrier between the porous Si shell and the solid Si core (Fig. S3).

Furthermore, photonic habituation was demonstrated in the NW device by illuminating the 658-nm laser on the porous shell region (Fig. 2d, e). We measured current levels under a bias voltage of 5 V, while the laser was off and on for every 7 s (Fig. 2d). In the dark, the current increased from ~0.5 to ~6 μA by electrical charging. In contrast, when the laser was turned on, the high current level was immediately decreased to maintain the low current level, as expected in Fig. 1. In response to the laser cycle, the measured current level reproducibly cycled with the periodic dark and light conditions. Systematic experiments were performed under various illumination conditions with laser powers ranging from 17 to 720 μW. Notably, as the laser power increased, the current in the light condition decreased. This photon-triggered current level was plotted as a function of the pump power, revealing that the lowest laser power for photonic habituation was ~17 μW (Fig. 2e). In addition, when the laser power was larger than ~520 μW, the current was maintained at ~2.5 μA. Furthermore, we investigated current levels under laser illumination at various wavelengths (Fig. S4). The measurement showed that the current in the light condition decreased further as the wavelength of the pump laser was shorter. We note that photonic habituation is highly sensitive to laser power and wavelength, enabling the precise analog control of the current in the memory device.

**Fig. 3:**
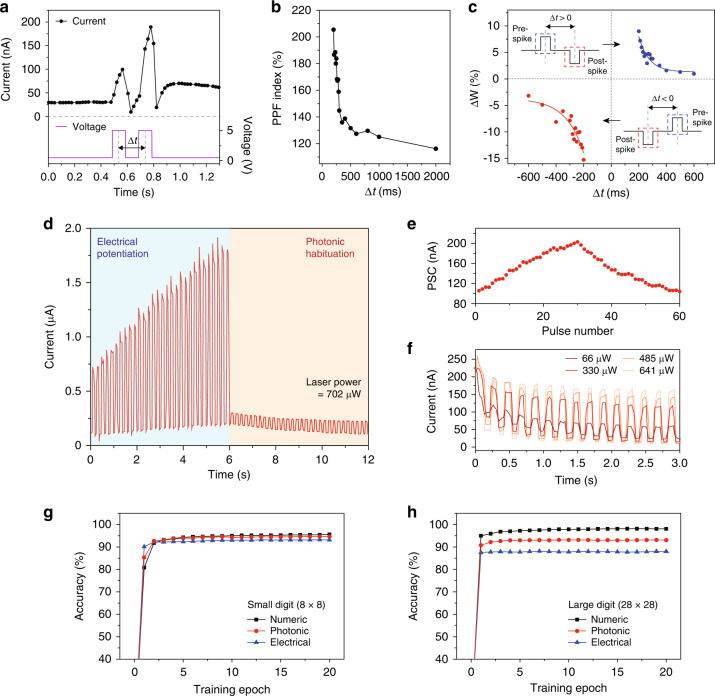
Synaptic behaviors. **a** Measured current (top panel) with applying a pair of voltage pulses (bottom panel). The current and voltage are compared on the same timeline. Δ*t* indicates the time interval between the two pulses. **b** PPF index plotted as a function of Δ*t*. **c** Relative synaptic weight, Δ*W*, as a function of the time interval between pre- and post-synaptic activities, Δ*t*. Δ*W* values for Δ*t* > 0 (blue dots) and Δ*t* < 0 (red dots) indicate the long-term potentiation and habituation, respectively, and are fitted with STDP learning functions (colored solid lines; see “Methods” section). **d** Measured current as a function of time, by applying 5 V voltage pulses for the first 6 s and by illuminating 658-nm laser pulses with a power of 702 μW (width of 100 ms and Δ*t* of 200 ms) for the next 6 s. The read voltage was 0.5 V. The device has a different channel length than the one in (**a**). **e** PSC as a function of the pulse number. **f** Measured current as a function of time by laser pulses with different powers, following the potentiation process for 6 s. The laser powers were 66, 330, 485, and 641 μW. The other parameters were the same as those in (**d**). **g**, **h** Simulated backpropagation training accuracies for 8 × 8 pixels (**g**) and 28 × 28 pixels (**h**) handwritten digit images. The graphs for the ideal numeric (black), photonic (red), and electrical processes (blue) are plotted as a function of training epoch. The experimentally derived parameters shown in Figs. 3e and S9 were used for the photonic and electrical cases, respectively (see “Methods” section)

By using the memory behavior and photonic habituation, the NW can function as an artificial synaptic device^8,12^. To examine the synaptic behaviors of short-term and long-term plasticity, we measured the current by applying paired pulses with a peak voltage of 5 V, a width of 100 ms, and a time interval (Δ*t*) of 200 ms (Fig. 3a). The current level was then recorded with a read voltage of 0.5 V; the operation of the NW device was not affected by this small read voltage. The measurement showed that two current peaks were generated by the voltage pulses. The excitatory postsynaptic current (EPSC), the difference between the next peak current and the initial current, increased with each voltage pulse^12^. We can understand these features based on our model in Fig. 1: a current percolation path is created inside the nanograin network by the electrical charging of the nanograin when a voltage pulse is applied. When two short-interval pulses are applied, the formation of percolation paths overlaps, resulting in a higher second current peak than the first one. The on-off ratio for the first voltage pulse in Fig. 3a was ~4, which is comparable to previous work^8,33,34^. Furthermore, the enhanced current called postsynaptic current (PSC) was observed after the voltage pulses were turned off. In particular, the PSC decreased slowly with increasing time and exhibited the significant dependence on the iteration number of the voltage pulses and the width of the single voltage pulse (Fig. S5). We note that these behaviors of the NW device are similar to the plasticity properties of synapses^12,35^.

To assess the characteristics of the short-term plasticity, we obtained the paired-pulse facilitation (PPF) index defined by the difference between the first and second EPSCs, with varying Δ*t* from 200 to 2000 ms (Fig. 3b). The maximum PPF was 205% at Δ*t* of 200 ms, and this value decreased gradually as Δ*t* increased. Such a high PPF index of the short-term synaptic plasticity enables the demonstration of volatile memory devices^35^. In addition, the spike-timing-dependent plasticity (STDP) was investigated to show the long-term plasticity for a non-volatile memory behavior^12,13^. To demonstrate the STDP, the ratio of the PSC and initial current level, Δ*W*, was plotted as a function of Δ*t* (Fig. 3c). A pair of the +5 and –5 V (or –5 and +5 V) voltage pulses were applied for Δ*t* > 0 (or Δ*t* < 0), to realize the cases that a pre-synaptic spike leads to (or follows) a post-synaptic spike (insets, Fig. 3c). In both cases, a shorter Δ*t* resulted in greater values of the PPF index and Δ*W* on the device, enabling the modification of short-term and long-term memory, respectively. When the pulse interval is sufficiently long, the increase rates of the PPF index and Δ*W* are low due to the loss of some charge between each pulse.

Next, we demonstrated electrical potentiation and photonic habituation for memory and erasure, respectively, using the plasticity property of the NW device (Fig. 3d). First, the potentiation process was performed by applying voltage pulses of 5 V (width of 100 ms and Δ*t* of 200 ms) for the first 6 s, which resulted in the gradual increase of PSC. The NW device was reliable in the five cyclic potentiation processes (Fig. S6). After the potentiation was done, the photonic habituation was performed for the next 6 s. In this process, the 658-nm laser pulses (power of 702 μW, width of 100 ms, and Δ*t* of 200 ms) were incident to the NW shell, while the current was recorded by the read voltage of 0.5 V (Fig. S7). The measurement showed that the current level was modulated depending on the on and off states of the laser. Interestingly, the current increased and decreased rapidly in the light and dark conditions, respectively. This process is different from the one in Fig. 2d, which showed the reduction in current in the light condition due to the application of a continuous bias voltage of 5 V to the NW.

For the quantitative analysis of the photonic habituation, we plotted the PSC as a function of the pulse number (Fig. 3e). The PSC gradually decreased during 30 repetitions of the laser pulse. Whereas the maximum PSC was ~200 nA at the 30th voltage pulse in the potentiation process, the PSC decreased linearly with increasing number of laser pulse, down to ~100 nA at the 60th laser pulse. We also analyzed the current levels during photonic habituation with varying laser powers from 66 to 641 μW (Fig. 3f). We measured the higher (lower) current in each light (dark) condition, as the laser power increased, which resulted in a smaller PSC (Fig. S8). The consecutive photonic habituation of the electric charges stored in the porous NW shell during potentiation can explain the decrease in PSC with increasing number of laser pulse. In this case, higher laser power releases more stored charges and reduces the PSC further.

Furthermore, we performed the pattern recognition simulations to investigate the potential of the NW devices for neuromorphic computing, when photonic and electrical habituation processes were used. To compare photonic and electrical habituation, we also demonstrated electrical habituation following electrical potentiation (Fig. S9). The current level modulation and PSC behavior were similar to those shown in Fig. 3d, e, respectively; however, the linearity of the PSC was lower than in Fig. 3e. According to our model, in photonic habituation, the charged electrons in the nanograin network are excited to a higher electronic state and are immediately removed by the illumination^20^, resulting in a linear decrease of the PSC with increasing number of laser pulses (Fig. 3d, f). However, in electrical habituation, the linearity of the PSC becomes relatively poor due to the Schottky junction between the porous Si shell and the solid Si core^36^ (Fig. S9). Based on the PSC fitting results obtained in Figs. 3e and S9, the backpropagation method, a common method for benchmarking synaptic array architectures, were used on two data sets of 8 × 8 and 28 × 28 pixels image versions of handwritten digits^37^. We calculated the recognition accuracies of networks after training epochs (Fig. 3g, h). After 20 training epochs, the photonic and electrical processes can read small-size (large-size) handwritten digits with accuracies of 94.7% (93.1%) and 93.2% (88.0%), respectively, showing that the photonic process approaches the ideal case with an accuracy of 95.5% (98.1%). Therefore, these comprehensive results confirm that the NW memory functions as a reliable synaptic device. Furthermore, the large difference in numeric, photonic, and electrical accuracies for large-size handwritten digits compared to small-size handwritten digits is due to the fact that networks processing large-size handwritten digits have more synapses than networks processing small-size handwritten digits, making them more vulnerable to the nonlinear properties of the device.

**Fig. 4:**
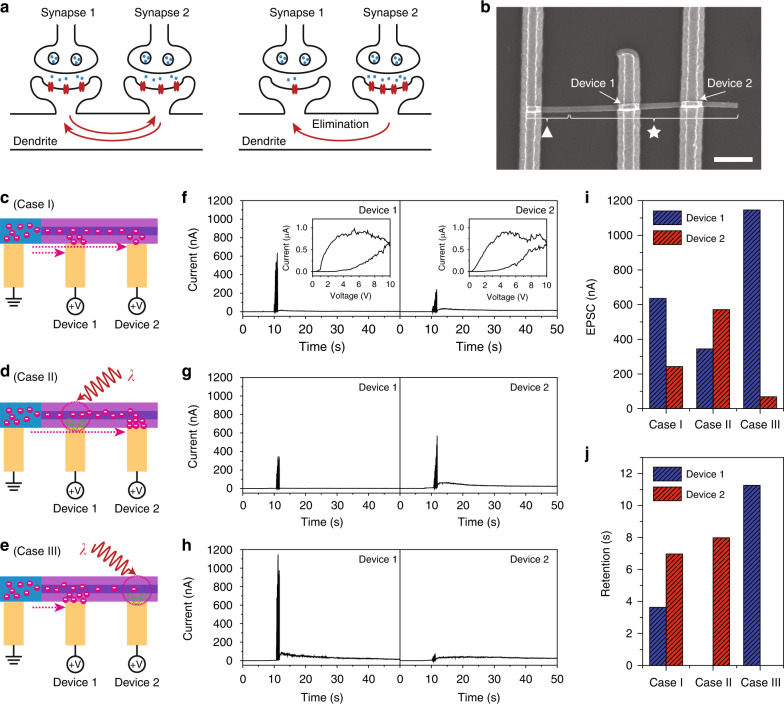
Synaptic elimination using two adjacent NW devices. **a** Schematic illustration of synaptic elimination occurring between two adjacent synapses. **b** SEM image of two fabricated devices in a single NW. Symbols of ▲ and ★ indicate the pure solid and core/shell segments, respectively. Both devices 1 and 2 were fabricated on the core/shell segment (★), whereas the ground electrode was fabricated on the pure solid segment (▲). Scale bar, 2 μm. **c**–**e** Schematic illustrations showing the operations of device 1 and device 2 in the dark condition (**c**: Case I) and under illumination on either device 1 (**d**: Case II) or device 2 (**e**: Case III). **f**–**h** Measured currents in device 1 (left panel) and device 2 (right panel) as a function of time, for Case I (**f**), Case II (**g**), and Case III (**h**). For the potentiation process, 10 electrical pulses with a peak voltage of 5 V, a width of 100 ms, and Δ*t* of 200 ms were applied between 10 and 12 s. The 658-nm laser with a power of 2.1 mW was incident continuously to either device 1 (**g**) or device 2 (**h**). The read voltage was 0.5 V. The insets in the left and right panels of (**f**) show the *I*–*V* curves of devices 1 and 2, respectively. **i**, **j** EPSC (**i**) and retention (**j**) changes of devices 1 and 2 for Case I, Case II, and Case III. The EPSC of device 1 was initially ~640 nA (Case I) and changed to ~340 nA (Case II) and ~1150 nA (Case III). Also, the EPSC of device 2 was changed to ~570 nA (Case II) and ~70 nA (Case III) from ~240 nA (Case I)

Finally, we highlight that using our NW devices, we can demonstrate a new synaptic interaction that is not achievable in electrical processes. For example, in two adjacent NW devices connected in parallel, photonic habituation as the kill switch prevents the construction of a current percolation path of one device and allows the enhancement of current in the other device. In fact, this process mimics the synaptic elimination in biological systems^38^. While two synapses are active with balanced stimulations (left, Fig. 4a), punishment signals from the more active synapse (synapse 2) can inactivate the less active synapse (synapse 1) (right, Fig. 4a). Since the illumination on one NW device during the potentiation process functions as a punishment signal, the other NW device in the dark condition exhibits the enhanced signal. Such a process cannot be demonstrated using electrical habituation, because the current percolation path is always formed by applying a forward or reverse bias voltage.

Two adjacent NW devices were fabricated in a single NW, using a long core/shell segment and two metal electrodes on it with 2 μm distance (Fig. 4b). Device 1 and device 2 defined by the two electrodes are connected in parallel by the solid core with low resistance. The insets of Fig. 4f show the measured *I*–*V* curves of the two NW devices; similar hysteresis loops were observed. Then, using these devices, we design three different cases to emulate various synaptic interactions. First, in Case I, the two adjacent devices are in the dark condition (Fig. 4c). The charges move through the solid core, and the current percolation paths are generated in both devices. Second, in Case II, the laser is illuminated on only device 1 (Fig. 4d). Device 1 has a relatively high resistance since the current percolation path cannot be created there, and more charges move toward device 2. Third, Case III is similar to Case II, but device 2 is under illumination and only device 1 exhibits the high current flow (Fig. 4e).

These three cases were examined through systematic experiments (Fig. 4f–h and “Methods” section). For the potentiation process, 10 pulses with a peak voltage of 5 V, a width of 100 ms, and Δ*t* of 200 ms were applied to both the devices between 10 and 12 s. The current of each device was measured as a function of time. While Case I was realized in the dark condition, the 658-nm laser with a sufficiently high power was continuously illuminated on either device 1 or device 2 for Case II or Case III, respectively. In the dark condition of Case I, the current of device 1 was measured to be larger than that of device 2, because the solid channel length for device 2 was longer than that of device 1 (Fig. 4f). In contrast, in Cases II and III, the current in the device illuminated by the laser decreased almost twice, whereas the current in the other device increased twice (Fig. 4g, h). For the quantitative analysis, we summarized the EPSC and retention in the three cases (Fig. 4i, j). In particular, the retention of the devices eliminated by the light illumination (Fig. 4j) can be explained by the annihilation of charges in the porous shell. Therefore, the synaptic elimination was verified in terms of the current level and retention of each device. Taken together, this synaptic interaction is a successful example of providing groundbreaking application possibilities by leveraging the photonic features of the NW memory device.

## Discussion

In summary, we developed a nanograin network memory using reconfigurable percolation paths in a single Si NW with the solid core/porous shell and pure solid core segments. The electrical and photonic control of current percolation paths demonstrated the analog and reversible adjustment of the persistent current level. The NW device exhibited memory behavior with a hysteresis loop and current suppression using photonic habituation. In addition, the synaptic behaviors of memory and erasure were demonstrated by performing the potentiation and habituation processes in the single NW memory device. Notably, photonic habituation was sensitive to the incident laser power, showing a linear decrease in the PSC. Furthermore, photonic manipulation of the potentiation processes in two adjacent devices interconnected on a single NW enabled mimicking of the synaptic elimination. Therefore, photonic habituation in a NW memory device can show various synaptic interactions with a new function such as the kill switch.

Overall, electrical and photonic reconfiguration of conductive paths in Si nanograin networks opens up a new paradigm for next-generation nanodevice technologies. For a more practical implementation, the single NW memory device needs to address the following issues. First, while our NW devices are operated by electrical potentiation and photonic habituation, all-optical operation is necessary to fully address the endurance issue. Since photonic potentiation, as opposed to photonic habituation, is already present in other devices^25,26,39^, all-optical NW devices capable of both photonic habituation and potentiation can be developed by combining unique features of these devices. Second, scaling up the NW device is required for the use of a practical synaptic device. To this end, two-step metal-assisted chemical etching^40^ and nanocombing assembly techniques^41^ can be used to fabricate porous segments at desired locations in a NW and aligned NW arrays, respectively. In the future, using the single NW memory device, it will be interesting to explore synaptic devices with synaptic density and energy efficiency comparable to the human brain^42,43^, because smaller device sizes reduce power consumption while increasing integration density.

## Methods

### Transport model in the porous shell

We theoretically analyze the electric current in the porous shell using two different transport mechanisms: (1) electron hopping and (2) space-charge-limited currents. First, we consider the electron hopping current. Because of the self-capacitive nature, the electrons can be localized in the Si nano-crystalline grains of the porous shell^29,44^. These electrons move to the neighboring grains by hopping over the Coulomb barrier. Then, the current due to electron hopping is given by

1 $${I}_{H}={\sigma }_{0}\exp \left(-\frac{e{E}_{A}}{{k}_{B}T}\right)\exp \left(\sqrt{\frac{{V}_{d}}{{V}_{0}}}\right){V}_{d}$$

where *e*, *k*_*B*_, *T*, *σ*_*0*_, *E*, and *V*_*d*_ are the elementary electric charge, Boltzmann constant, temperature, conductivity prefactor, activation energy, and bias voltage from drain to source, respectively^29,32^. Here, *V*_*0*_ is given by

2 $${V}_{0}={k}_{B}^{2}{T}^{2}{e}^{-2}{\left(\frac{e}{\pi {\varepsilon }_{0}{\varepsilon }_{r}d}\right)}^{-1}$$

where *ε*_*0*_, *ε*_*r*_, and *d* are the permittivity in vacuum, dielectric constant of Si, and thickness of the porous shell, respectively^44^. Because of the high resistance of hopping transport, *I*_*H*_ is low even for porous layers with tens of nanometers in thickness^44^.

Next, we consider the space-charge-limited current. Due to the charging in the nano-crystalline grains, continuous networks of charged grains can form current percolation paths, allowing for the space-charge-limited current (*I*_*SCL*_)^32,45^. With the analogy of the conventional field effect transistor model, *I*_*SCL*_ is given by

3 $${I}_{{SCL}}=\frac{{\mu }_{{eff}}{C}_{{grain}}}{d}\left({V}_{g}-{V}_{{TH}}-\frac{{V}_{d}}{2}\right){V}_{d}$$

where *C*_*grain*_, *μ*_*eff*_, *d*, *V*_*g*_, and *V*_*TH*_ are the self-capacitance of a grain in unit length, effective mobility, thickness of the porous shell, effective gate voltage, and threshold voltage, respectively. *I*_*SCL*_ is much higher than *I*_*H*_, due to the current percolation paths from source to drain.

The contribution of *I*_*H*_ and *I*_*SCL*_ to the total current varies depending on charging. We examine the change in total current using the percolation theory of conductivity^32^. The stored charge in the porous shell is described by the parametric charge, *Q*_*P*_. In addition, the contribution of *I*_*SCL*_ to the total current is described using the weight function, *w* = (*Q*_*P*_ – *Q*_*C*_)^*α*^, which indicates the number of current percolation paths. Here, *Q*_*C*_ and *α* are the critical parametric charge and the critical percolation conductivity exponent, respectively. When *Q*_*P*_ < *Q*_*C*_, *w* is zero because there are no current percolation paths. In Fig. 1c, *w* was calculated with *Q*_*C*_ of 0.2 and *α* of 1.3. The total current, *I*_*D*_, is given by

4 $${I}_{D}=\left(1-w\right){I}_{H}+w{I}_{{SCL}}$$

Next, we analyze the change in *I*_*D*_ as a function of the applied voltage, *V*_*D*_. *I*_*D*_ is a function of *Q*_*P*_, and *Q*_*P*_ is given by

5 $${Q}_{p}\left({t}_{0}+\varDelta t\right)={Q}_{p,0}+\left[{V}_{D}{C}_{{grain}}-{Q}_{p,0}\right]\left[1-\exp \left(-\frac{\varDelta t}{\tau }\right)\right]$$

where Δ*t*, *τ*, and *Q*_*p*,*0*_ are the charging time interval, characteristic time, and parametric charge at time *t*_*0*_, respectively. The porous shell is assumed to be a thin-layer capacitor. The constant *V*_*D*_ is applied for Δ*t* at *t*_0_. The time series of *Q*_*P*_ is determined iteratively with the history of the applied *V*_*D*_. To investigate the electric current in the porous shell, we introduce the physical parameters of porous Si to calculate the hysteresis loop in the *I*_*D*_ – *V*_*D*_ curve (Fig. 1e). *σ*_*0*_, *d*, *E*_*A*_, and *ε*_*r*_ are set to 0.01 Ω^−1^ m^−1^, 20 nm, 0.32 eV, and 11.7, respectively. *C*_*grain*_ is set to 6.5 × 10^−10^ C V^−1^ m^−1^ using the self-capacitance of a sphere with a diameter of 3 nm. Also, *μ*_*eff*_ is set to 1 cm^2^ V^−1^ s^−1^, which is similar to the value of amorphous Si^46,47^. In addition, we set *τ*, *V*_*g*_, *V*_*TH*_, *α*, and *Q*_*c*_ to 1 s, 2.5 V, 0.5 V, 1.3, and 0.2, respectively, as the free parameters. Then, in the forward (backward) *V*_*D*_ sweep from 0 to 5 V (from 5 to 0 V), the voltage step of 0.1 V (–0.1 V) is used. The constant *V*_*D*_ is applied during Δ*t* of 70 ms in each voltage step. *Q*_*p*,*0*_ is set to zero at *t* = 0. With these parameters, *I*_*D*_ and *Q*_*P*_ are calculated using Eqs. (4) and (5) (Fig. 1e).

### Device fabrication

Si NWs with solid core/porous shell and pure solid core segments were fabricated using a metal-assisted chemical etching (MaCE) method^20,48^. First, a monolayer of hexagonal-lattice closely packed polystyrene (PS) beads was transferred to the surface of an n-Si substrate with a moderated doping level (1–10 Ω·cm). The diameters of the PS beads were reduced from 300 nm to 180 nm by O_2_ plasma etching with a power of 50 mW for 20 s. Next, a 50-nm-thick Au layer was deposited on the prepared sample using thermal evaporation, resulting in the formation of Au mesh on the Si substrate. The PS beads were removed by sonication in ethanol. To fabricate the NW shown in Fig. 2b, the Si substrate with the Au mesh was immersed in a mixed solution of HF, H_2_O_2_, and H_2_O (volume ratio is 5:1:6) at room temperature for 10 min. A porous shell was formed on the NW surface, by applying an external pulsed voltage of 4.5 V for 10 s to the Au mesh during the etching process. To fabricate the NW shown in Fig. 4b, two steps of MaCE were conducted. First, the Si substrate with the Au mesh was immersed in a mixed solution of HF, H_2_O_2_, and H_2_O (volume ratio is 5:0.5:3) at room temperature for 2 min, to generate a short solid segment. Then, the sample was immersed in a mixed solution of HF, H_2_O_2_, and H_2_O (volume ratio is 5:1.5:3) at room temperature for 5 min, to generate a long core/shell segment. Finally, to fabricate the NW memory device, the prepared NWs were dispersed onto a Si_3_N_4_/SiO_2_/Si substrate, and metal contacts were defined using aligned electron-beam lithography and thermal evaporation of Ti/Au (7/300 nm). A lift-off process was conducted by immersing the sample in acetone for 1 h.

### Electrical measurements

The *I*–*V* curves of the Si NW devices were measured using a source measure unit (2450 SourceMeter, Keithley) and a customized probe station. Voltage pulses were generated using a wavefunction generator (33500B, Keysight), and the current was measured as a function of time using a multimeter (DMM7510, Keithley). In the STDP experiment, the post-spike has the opposite polarity of potential to the pre-spike, and thus, it is commonly used to apply two bias voltages of opposite polarity to the same electrode to achieve pre-spike and post-spike^12,13^. The PPF index and Δ*W* were recorded as the average of five measurements for each experiment. The PPF index was defined as (*I*_*1*_ – *I*_*i*_)/(*I*_*2*_ – *I*_*i*_) × 100%, where *I*_*i*_, *I*_*1*_, and *I*_*2*_ are the currents before the input spike voltage, current of the first spike voltage, and current of the second spike voltage, respectively. Δ*W* was defined as (*W*_*t*_ *–* *W*_*0*_)/*W*_*0*_ × 100%, where *W*_*t*_ and *W*_*0*_ are the currents after and before applying the paired voltage pulses, respectively. After measuring each data point of the PPF index and Δ*W*, the low resistance state was reset by applying a negative voltage of –1 V to the NW device for 5 s.

### Optical measurements

Photonic habituation were demonstrated using the optical measurement setup shown in Fig. S7. The pump laser diode (LD) with a wavelength of 658 nm was focused on the NW devices using a ×50 objective lens with a numerical aperture of 0.55. A supercontinuum laser with a wide wavelength range of 480 to 760 nm (SuperK EXTREME EXB-4, NKT Photonics) was used in Fig. S4. The spot size of the laser was ~1 μm. In Figs. 2d, 3d–f, and 4, a pulsed laser (repetition rate: 1 MHz, pulse width: 10 ns) was used to minimize the thermal effect in the NW devices.

### STDP and retention fitting

The values of Δ*W* in Fig. 3c were fitted using an STDP learning function,

$$\triangle W=\,{A}_{+}{{\exp }}\left(\frac{-\triangle t}{{\tau }_{+}}\right)\,{\rm{if}}\,\triangle t \,> \,0,\,{\rm{and}}\,{A}_{-}{{\exp }}\left(\frac{-\triangle t}{{\tau }_{-}}\right)\,{\rm{if}}\,\triangle t \,<\, 0$$

The linear factors ${A}_{+}$ and ${A}_{-}$, which indicate the maximum change in device resistance for a single switching event, were obtained as 190.34 and 121.62, respectively. The exponential parameters ${\tau }_{+}$ and ${\tau }_{-}$, which represent the learning rate of the synapse, were obtained as 154.8 ms and 120.8 ms, respectively.

The retention in Fig. 4j was obtained by fitting the currents in Fig. 4f–h using an exponential decay function,

$${A}_{1}{{\exp }}\left(\frac{-t}{\tau }\right)+{y}_{0}$$

where ${y}_{0}$ is the current of memory at stabilized state, *A*_1_ is the prefactor, and *τ* is the relaxation time constant.

### Pattern recognition simulations

We performed an artificial neural network simulation based on the platform CrossSim^42,49,50^, using the experimentally measured PSC characteristics. A three-layer (one hidden layer) neural network was used for the supervised learning with backpropagation. The network simulations were performed on two data sets: a small image version (8 × 8 pixels) of handwritten digits from the “Optical Recognition of Handwritten Digits” dataset and a large image version (28 × 28 pixels) of handwritten digits from Modified National Institute of Standards and Technology (MNIST) dataset^51^. We trained our network using the backpropagation algorithm with a gradient descent function. For small digit images, the network size was 64 × 36 × 10. After training with 3823 images, recognition was performed on a 1797-image testing set that had not been used for training. For large digit images, the network size was 784 × 300 × 10. After training with 60,000-images, recognition was performed using a separate 10,000-image testing set. The evaluation of recognition accuracy was repeated 20 times (20 epochs).

Totally symmetric and linear PSC changes were used for an ideal weight update process. However, the PSC features measured in our experiment were asymmetric and nonlinear. To include the nonlinearity of PSC in the simulation, we used the following equations for conductance ($G={I}_{{PSC}}/{V}_{{read}}$), which changes as a function of the normalized pulse number *P*^50^:

$$G={G}_{1}\left(1-{e}^{-\nu P}\right)+{G}_{\min }\,({\rm{Positive\; pulse}})$$

$$G={G}_{\max }-{G}_{1}\left(1-{e}^{-\nu (1-P)}\right)\,({\rm{Negative\; pulse}})$$

$${\rm{where}}\,{G}_{1}=\,\frac{{G}_{\max }-{G}_{\min }}{1-{e}^{-\nu }}$$

Here, *G*_min_ and *G*_max_ are the minimum and maximum conductance, respectively, and *ν* is a parameter that characterizes the nonlinearity of the conductance. The response is exactly linear when $\nu =0$. In our case, the PSC values between 21 and 72% for electrical habituation and 10 to 66% for photonic habituation were used to extract the nonlinearity parameters. Then, *ν* were 0.9 and 0.37 for the electrical and photonic habituation, respectively. Based on the built-in asymmetric nonlinear update model of CrossSim, these values were used in the simulation process^51^. In addition, a learning rate of 0.1 was used to simulate small-digit and large-digit images.

## Supplementary information

Supplementary Information

## Data Availability

The data that support the findings of this study are available from the corresponding authors upon reasonable request.
