# Supplementary material for: Nanograin network memory with reconfigurable percolation paths for synaptic interactions
Source: Light Sci Appl. 2023 May 15;12:118. doi: 10.1038/s41377-023-01168-5 (PMC10185519; doi:10.1038/s41377-023-01168-5)
Supplement: Supplementary file 1 — Supplementary Information [file 41377_2023_1168_MOESM1_ESM.docx]

**Supplementary Information for**

Nanograin network memory with reconfigurable percolation paths for synaptic interactions

Hoo-Cheol Lee^1^†, Jungkil Kim^2^†*, Ha-Reem Kim^1^†, Kyoung-Ho Kim^3^†, Kyung-Jun Park^1^, Jae-Pil So^1^, Jung Min Lee^1^, Min-Soo Hwang^1^, and Hong-Gyu Park^1^*

*Correspondence to: jungkil@jejunu.ac.kr; hgpark@korea.ac.kr

†These authors contributed equally to this work.

Figures S1–S9

Table S1

**Supplementary Figures**

**
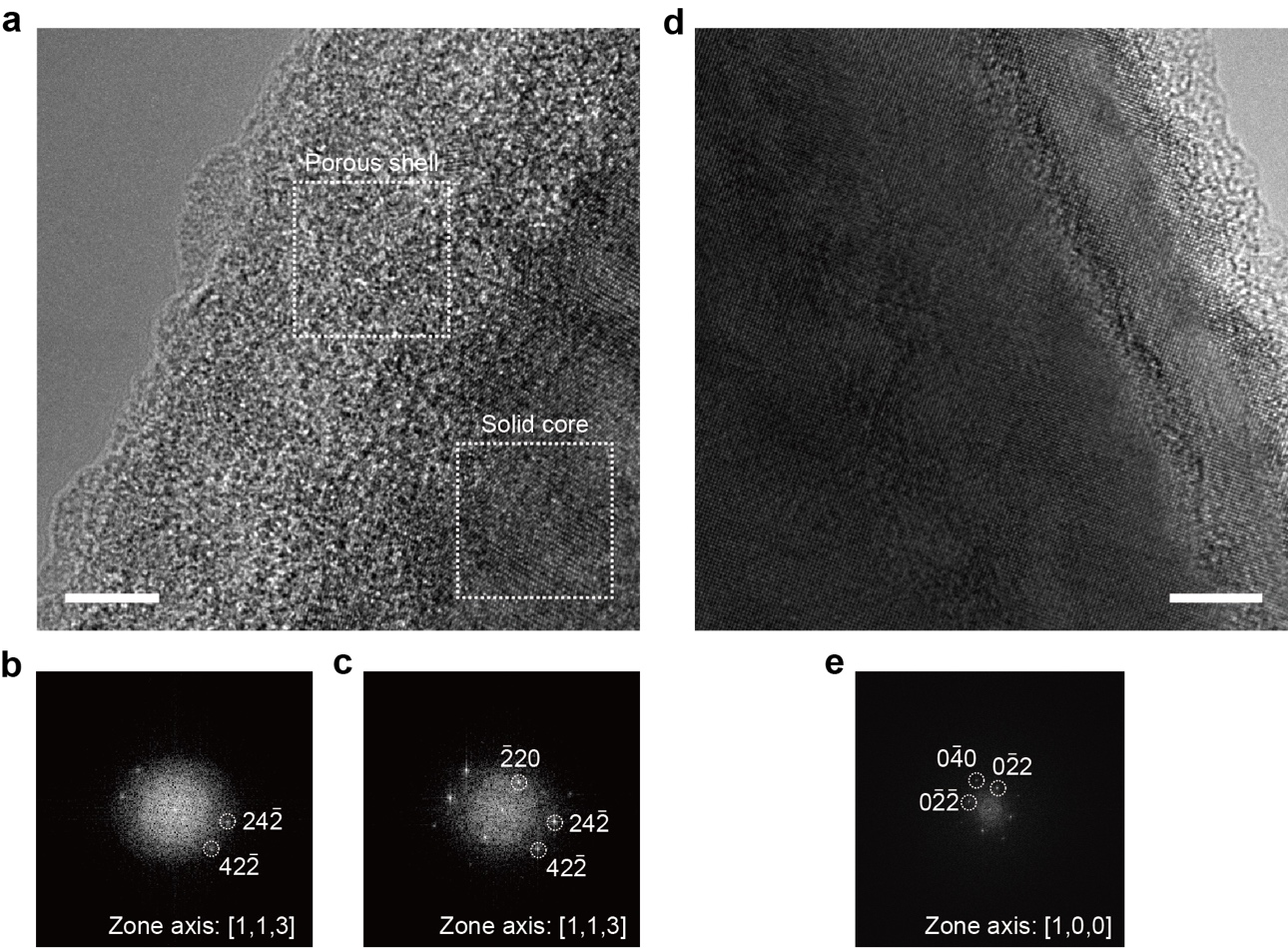
**

Fig. S1. TEM analysis. a, High-resolution TEM image of a core/shell segment of the fabricated Si NW. A number of nano-crystallites and nanovoids were observed in the ~15 nm-thick porous shell. b and c, Fast Fourier Transform (FFT) patterns of the porous shell (b) and solid core (c) regions in white dash boxes in (a). The typical FFT pattern of Si was observed in the zone axis [113]. The FFT patterns of the porous shell show the same spots as that of the solid core, indicating that the porous shell has the same crystal lattice as the solid core. d, High-resolution TEM image of a long solid segment of the fabricated Si NW. e, FFT pattern corresponding to (d). The zone axis is [100]. The porous shell in (a) has a rougher surface than the solid segment in (d).


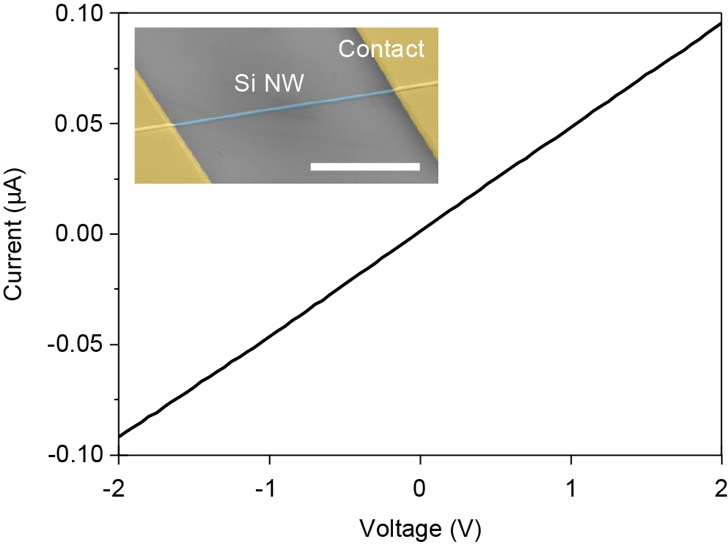


**Fig. S2. A pure Si NW device.** Measured *I*–*V* curve of a pure Si NW device. The diameter and length of the NW are 200 nm and 10 μm, respectively. Inset, SEM image of the device. Scale bar, 5 μm.


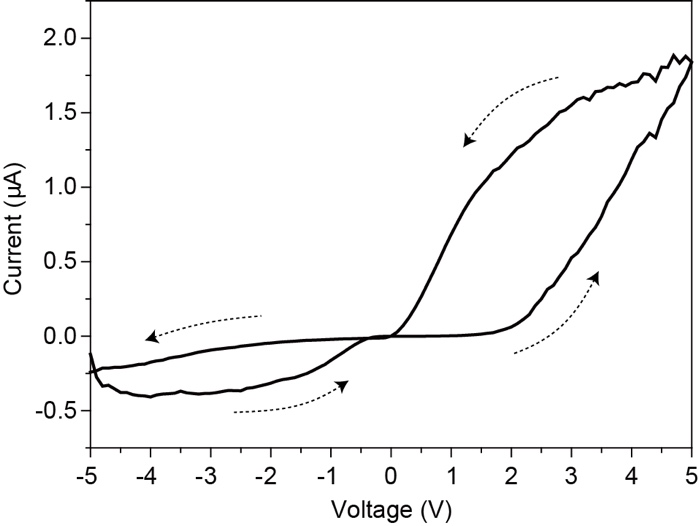


Fig. S3. Hysteresis loop of the NW memory device. Measured *I*–*V* curve with a dual sweep mode. The black dotted arrows indicate the sweep directions. This graph is the same as that in Fig. 2c, but it now contains the negative voltage sweep. The lower current level was formed in the negative sweep direction, because of the Schottky barrier between the porous Si shell and the solid Si core^44^.


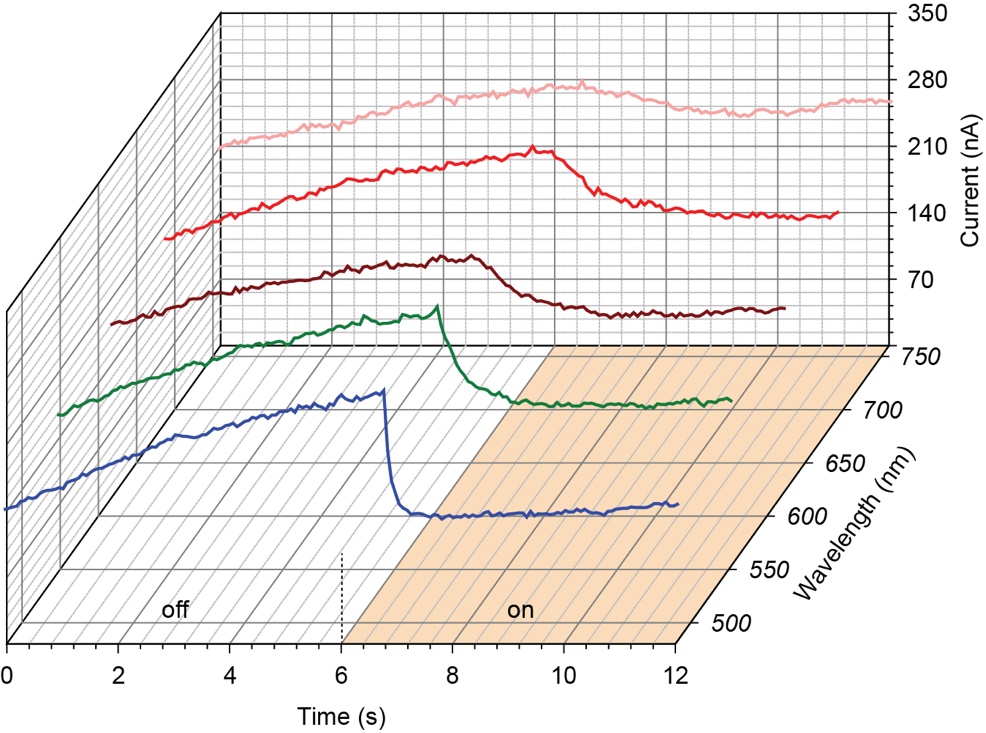


**Fig. S4. Wavelength-dependent response of a NW device.** Measured currents as a function of time, under the laser illumination with wavelengths of 480 (blue), 550 (green), 620 (brown), 690 (red), and 760 nm (pink). The laser power was 325 μW and the bias voltage was 5 V. The pump laser was off and on for 6 s each.


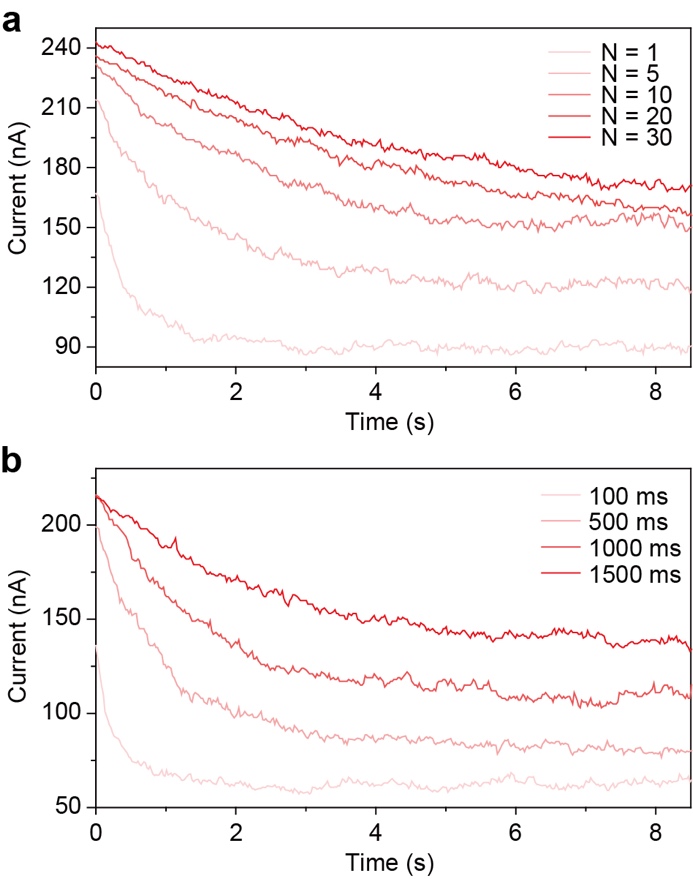


Fig. S5. PSC measurements conducted using voltage pulses with varying iteration numbers and widths. a, Measured PSCs as a function of time by applying 5 V pulses with different iteration numbers (*N*): *N* = 1, 5, 10, 20, and 30. The pulse width and Δ*t* were set to 100 ms and 200 ms, respectively. The PSCs were stabilized at ~90, ~120, ~150, and ~ 170 nA for *N* = 1, 5, 10, 20, and 30, respectively. b, Measured PSCs as a function of time by applying 5 V single pulse (*N* = 1) with different widths: 100, 500, 1000, and 1500 ms. The PSCs were stabilized at ~60, ~80, ~110, and ~135 nA for the widths of 100, 500, 1000, and 1500 ms, respectively.


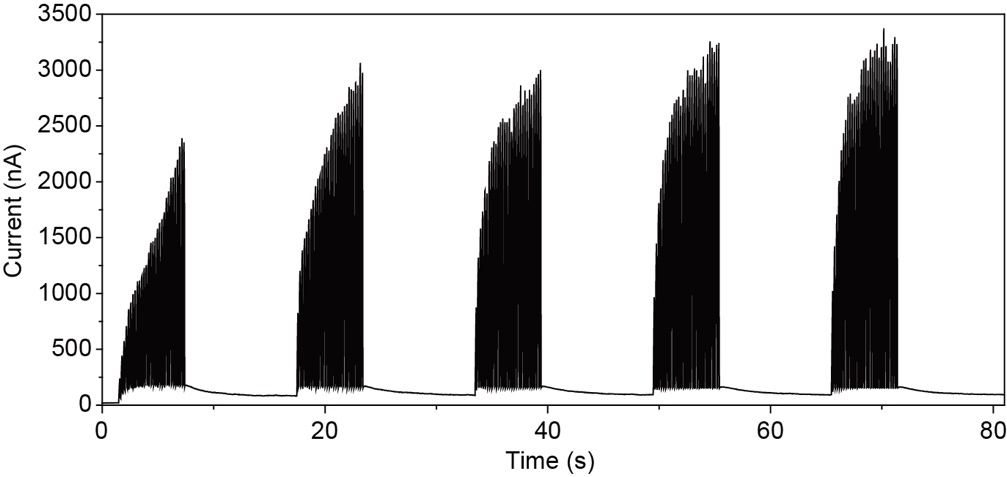


Fig. S6. Repeatable potentiation processes. Measured current as a function of time to show a repeatable potentiation process. In each potentiation, 5 V voltage pulses were applied 30 times with a width of 100 ms and Δ*t* of 200 ms for 6 s. The read voltage was 0.5 V. The initial current was ~20 nA from 0 to 1.5 s. The PSC was ~180 nA right after the first potentiation and ~85 nA right before the second potentiation. In the second potentiation, EPSC and PSC increased more rapidly than in the first one, because the current weight was formed in the first potentiation without habituation.


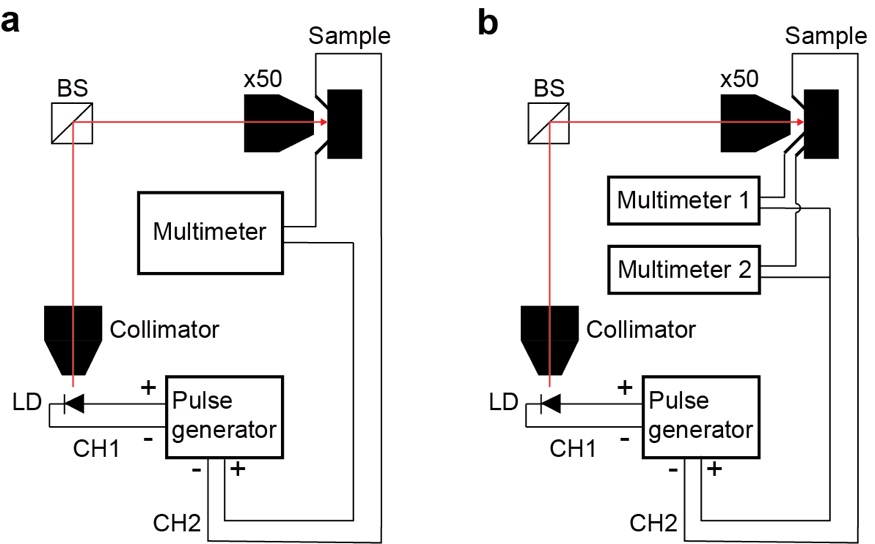


Fig. S7. Optical measurement setup. a, Experimental setup for Figs. 2d and 3d-f. A 658-nm laser diode (LD) was used to optically pump the NW devices mounted on a XYZ translation stage at room temperature. The LD spot size was reduced to ~1 μm using a ×50 objective lens with a numerical aperture of 0.55. The two-channel pulse generator was used to control the pump laser (CH1) and the bias voltage (CH2). The current in the NW device was measured using the multimeter connected in series. b, Experimental setup for Fig. 4. An additional multimeter (Multimeter 2) was added to the setup in (a), to measure the currents of two devices at the same time.


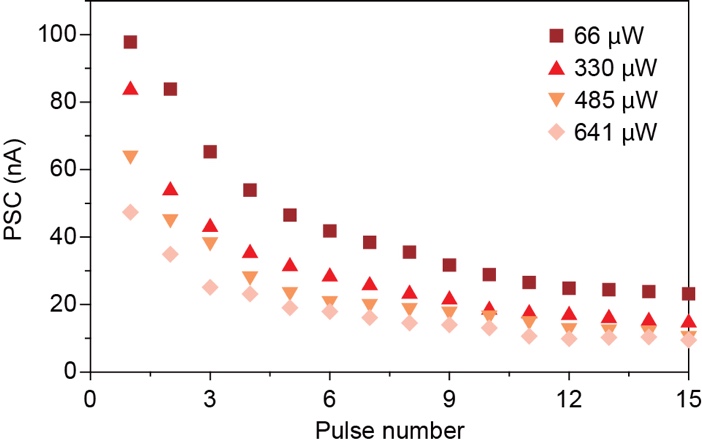


Fig. S8. PSCs of photonic habituation with various laser powers. Measured PSCs in Fig. 3f as a function of the pulse number for different laser powers.


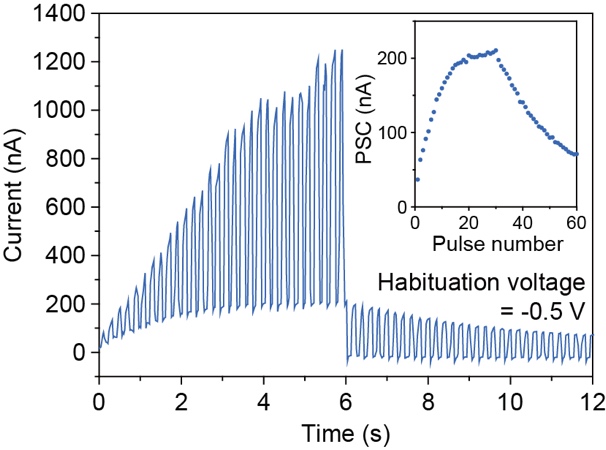


Fig. S9. Electrical potentiation and habituation. Measured current as a function of time, by applying 30 pulses with a positive peak voltage of 5 V for the first 6 s and 30 pulses of a negative peak voltage of –0.5 V for the next 6 s, for potentiation and habituation processes, respectively. The pulse width and Δ*t* were 100 ms and 200 ms, respectively. The read voltage was 0.5 V. Inset, PSC is plotted as a function of the pulse number. The maximum PSC was ~210 nA at the 30th voltage pulse, which were ~6 times larger than the first PSC of ~35 nA, whereas the PSC decreased to ~70 nA at the 60th voltage pulse.


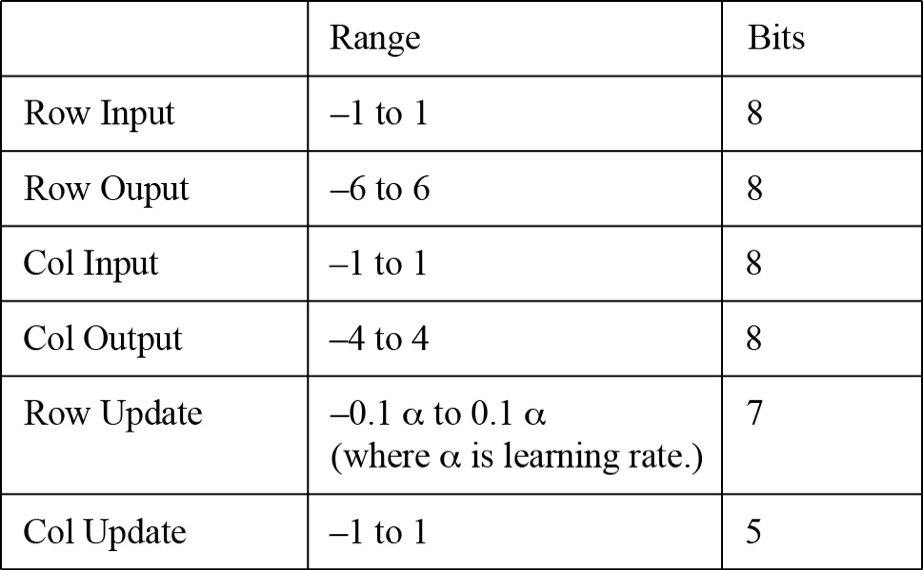


**Table S1. A/D and D/A parameters used in the simulations of backpropagation training accuracies.** We implemented each layer of the neural network using a simulated crossbar, which facilitated two critical operations: vector matrix multiplication and parallel rank 1 outer product updates. To enable analog-to-digital (A/D) and digital-to-analog (D/A) conversion, we followed the methods described in previous work^42,43^. The A/D and D/A parameters shown in the table allowed us to set the relevant parameters for the training process of Figs. 3g and 3h.

The synaptic weight is defined as the difference in conductance between two identical synaptic devices, denoted by *W* = *G*^+^ – *G*^–^. This approach enabled us to obtain positive and negative synaptic weights for use in our simulations. We simulated with synapse weights ranging from 0.1 to 1, after rescaling in accordance with the algorithm.
